# The C-Type Lectin Mincle: Clues for a Role in Crohn’s Disease Adjuvant Reaction

**DOI:** 10.3389/fimmu.2017.01304

**Published:** 2017-10-23

**Authors:** Anje A. te Velde

**Affiliations:** ^1^Tytgat Institute for Liver and Intestinal Research, Academic Medical Center, Amsterdam, Netherlands

**Keywords:** Mincle review, adjuvants, C-type lectin, *Mycobacterium*, Crohn’s disease

The term adjuvant is predominantly used when discussing vaccines, but only mimics how normally infections activate the immune system to secure that an innate immune reaction induces dendritic cells (DCs) to become optimally stimulatory for T cells. The interaction with the pathogen determines the different signals that are needed for a DC to become fully operated and give the proper polarizing factors to the differentiating T cell. Optimal co-stimulation requires a signal that is provided by upregulated receptors (CD80 and CD86) on DCs, and the T cell polarizing signal is mediated by various soluble or membrane-bound factors, like IL-12 for Th1 cell polarization. These signals are provided by ligation of pattern recognition receptors (PRRs), such as toll-like receptors (TLRs) and C-type lectins that can sense infection through recognition of pathogen-associated molecular patterns (PAMPs) or various inflammatory tissue factors (1).

Resting macrophages have low major histocompatibility complex class II and co-stimulatory molecules expressed on their surface. Macrophages can take up microorganisms *via* receptors such as scavenger receptors, complement receptors, and C-type lectins for degradation in phagosomes resulting in peptides for presentation. Macrophages also continuously scavenge dead or dying cells. These are a rich source of self-antigens, so it is very important that they do not activate naïve T cells when there is no ongoing microbial infection.

In Crohn’s disease (CD), a chronic inflammatory bowel disease (IBD), multiple factors have been described that contribute to disease pathogenesis (2). The exact etiology of IBDs still remains unknown, although it is thought that the diseases result from an excessive immune response directed against microbial or environmentally derived antigens that can be triggered by the disruption of the intestinal epithelial barrier integrity. The resulting inflammation is a very general reaction; a specific antigen mediating the inflammation has never been identified. The response is induced by the luminal microbiota, where microbial antigens as adjuvants stimulate the immune reaction. This results in activated innate (macrophages and neutrophils) and adaptive (Th1/Th17 and B lymphocytes) responses (3). In this respect, CD includes many characteristics of an immunologic adjuvant reaction.

Most proteins are poor immunogens when injected alone. Various substances that induce co-stimulatory, adjuvant, activity have been added in vaccines for a long time to induce appropriate antibody responses. Vaccines containing bacterial products, necessary for T cell responses, are very potent and therefore use in humans is limited. In recent years, the development of adjuvants that induce a strong cellular response has shifted from an empirical to a rational process based on knowledge of molecular mechanisms. A major breakthrough was the identification of the C-type lectin Mincle (macrophage-inducible C-type lectin) as one of the main receptors involved (4–6).

In this opinion article, I provide clues that the cellular adjuvant reaction that characterizes the pathophysiology of CD might be mediated by signaling *via* Mincle.

## Mincle

**Figure 1:**
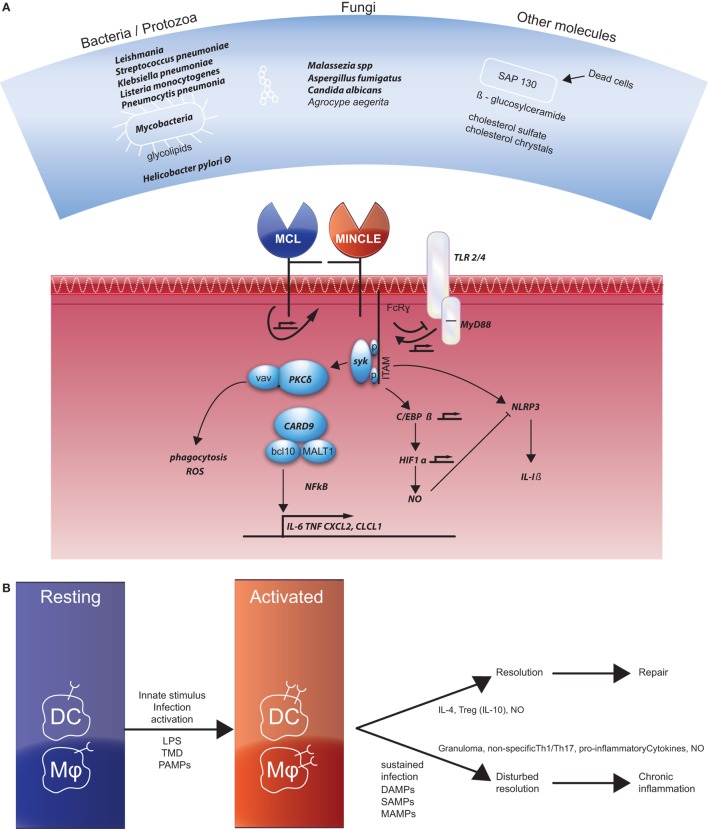
**(A)** Overview of the ligands of Mincle (top panel) and the signaling pathways described in the current literature. Factors that were also documented in literature related to Crohn’s disease are depicted in bold. References of the different ligands not mentioned in the text are for bacterial pathogen species (11–13), *Leishmania* (14), mycobacteria, and corynebacteria (15, 16), *Streptococcus pneumonia* (17), *Helicobacter pylori* (18), *Malassezia* (19, 20), *Aspergillus fumigatus* (21), *Candida albicans* (22, 23), SAP130 (10, 24, 25), β-glucosylceramide (26), cholesterol crystals (27), and cholesterol sulfate (28). References for the various signaling molecules not mentioned in the text are for TLR4/MyD88 (29), PKC (30), vav proteins (31), HIF1α (32) and Nlrp3 (33, 34). **(B)** Resting dendritic cells (DCs) and macrophages also do not express Mincle, but they do express macrophage C-type lectin (MCL). Different activating signals induce Mincle expression. Two different pathways induce repair or chronic inflammation as indicated. References for activation and chronic inflammation are given in the text, for the resolution: Th2 skewing *via* IL-4 (35, 36), IL-10 (37), and NO (38).

Mincle (also called clec4e or clecsf9) was first described as a downstream target of NF-IL6 (also named C/EBP-β), a transcription factor, in macrophages (7). They demonstrate that Mincle mRNA was strongly induced in response to several inflammatory stimuli, such as LPS, TNF-α, IL-6, and IFN-γ in murine macrophages. A few years later, Mincle was grouped together with macrophage C-type lectin (MCL), DC immunoreceptor, and Dectin-2 (DC-associated lectin-2) as type II-related C-type lectins (8). These genes were mapped in an arthritis susceptibility locus in a rat model and the first indication for an immune activating function of Mincle was proposed (9). Mincle serves as a receptor for various bacteria, fungi, and other molecules (listed in Figure 1A). Mincle signals *via* association with the FcRγ chain that contains an activating receptor coupled with an immunoreceptor tyrosine-based activation motif, ultimately resulting in activation of NF-κB (10).

The first suggestion that Mincle is a receptor for a cell wall component of *Mycobacterium tuberculosis*, the glycolipid trehalose-6,6′-dimycolate (TDM, also named cord factor), was made when it appeared to be involved in a characteristic process of mycobacterial infection: the formation of granulomas (4). Mycobacteria can persist in normal tissues (39); recruitment of Mincle by TDM coupled to immunoglobulin G-opsonized beads interferes with phagosome maturation (40). The activity of Mincle is mediated by a ligand binding site that is conserved in a wide range of mammalian species (41). The Th1/Th17 adjuvanticity of TDM and its synthetic analog trehalose-6,6′-dibehenate (TDB) including its molecular mechanism *via* Syk and Card9 was confirmed in several studies (5, 6, 42).

Mincle protein is barely detectable on resting cells (4, 42). In experiments in various tissues in rhesus macaques, the frequencies of CD14^+^ gated cells that express Mincle in colon and ileum were low compared with bone marrow, liver, spleen, and lymph nodes (43). Induction of Mincle expression was shown to be induced by several pathogenic and non-pathogenic stimuli. Mincle was shown to be induced by TDM in the absence of Mincle protein expression *via* MCL (also called dectin-3) that was constitutively expressed in myeloid cells (44–46) through protein–protein interaction *via* its stalk region (47). C/EBP-β is the central hub in Mincle expression and connects TLR4 signals to TDB/TDM responsiveness through MyD88-dependent upregulation of Mincle (32, 48).

## Mincle and CD

In literature, there is no direct link that connects Mincle to CD. There is, however, already a lot of information that links Mincle to other diseases. Most of these are also inflammatory-mediated diseases, such as rheumatoid arthritis (49, 50), allergic skin inflammation (28) and post-ischemic inflammation (51, 52), and other experimental inflammatory models (53–58).

Mincle has been shown to regulate numerous cellular responses including phagocytosis, endocytosis, respiratory burst, Nlrp3 inflammasome activation, NET formation, pro-inflammatory cytokine, and chemokine production and promotes Th1/Th17 responses [recently reviewed in Ref. (59, 60)]. These are all inflammatory reactions that have been described to play a role in CD. In Figure 1A, the different factors that are involved in Mincle signaling and also associated with CD are highlighted and discussed beneath.

As indicated, Mincle can act as a receptor for several different pathogens. The question is if these microorganisms have also been associated with CD. For *Mycobacterium avium* subspecies paratuberculosis, this is well known, it can be isolated from intestinal tissues and blood samples from CD patients at higher frequency than healthy persons (61). Treatment with antimycobacterial regimens in clinical trials achieved reversal of CD symptoms (62, 63). Also other bacteria linked to Mincle have been associated with CD: *Listeria monocytogenes, Klebsiella pneumonia, Streptococcus pneumonia, Pneumocystis pneumonia*, and *Escherichia coli* (64–70). A dysfunction in both a specialized form of autophagy, xenophagy, and HIF-1α was demonstrated to be involved in adherent invasive *E. coli* infections in CD (71). HIF-1α-induced inducible nitric oxide synthase produces nitric oxide (NO) that was shown to be upregulated in the inflamed mucosa in response to pro-inflammatory cytokines (72, 73).

In around 50% of CD patients’ granulomas can be detected (74). Granulomas are linked to mycobacterium, and Mincle has been shown to be important (75). Several cytokines, including IL-1 and TNF-α, have been shown to promote the formation of granulomas (76). These cytokines can be secreted upon stimulation *via* Mincle after stimulation with TDM-mediated granuloma formation (4).

Toward fungal glycans, it has been demonstrated that human peripheral blood mononuclear cells (PBMCs) from CD patients show a hyperresponsiveness with a central role for Syk and Src signaling (77). PBMCs from patients with CD produce more IFN-γ and IL-17 upon exposure to *Candida* (78).

A well-known complication of CD is intestinal fibrosis. Recently, it was demonstrated that this was mediated *via* a PKCδ-mediated redox-dependent signaling process by accumulated advanced oxidation protein products (79).

*CARD9* has been an autoimmune disease-associated gene, and differential expression of this gene might be a functional mechanism underlying observed GWAS signals (80). It coordinates Th17- and IL-22-producing cells in intestinal immune responses after epithelial injury in mice (81). Aberrant regulation of *CARD9*, either through genetic mutation (e.g., polymorphism) or activation by environmental triggers *via* Mincle, could contribute to pathological immune activation.

Crohn’s disease patients treated with TNF blockers demonstrated an increased risk of opportunistic infections such as mycosis, aspergillosis, pneumocystosis, or cryptococcosis (82) and also Pityrosporum (Malassezia) folliculitis (83), and cutaneous lesions of Leishmaniasis (84).

About 50% of the world’s population carry the *Helicobacter pylori* bacterium. In a meta-analysis, a negative association was found between *H. pylori* infection and CD. They conclude that *H. pylori* could exert an immunomodulatory effect in IBD (85) maybe by Mincle-mediated anti-inflammatory signaling (18).

Finally, danger/damage-associated molecular pattern (DAMP)-derived triggers from dead cells may contribute *via* Mincle to excessive and sustained inflammation in CD patients with active disease (86).

Taken together, numerous Mincle-related ligands and signaling molecules can be linked to CD.

## How Could Mincle Mediate CD Inflammation?

The efficacy by which Mincle handles microbes, microbial products, and damaged cells directs whether the outcome will be with suppression or with excess inflammation (see Figure 1B). These pathways can be polarized, but since CD is a chronic relapsing disease the activating signals and wound healing processes might also be present more or less in parallel. Mincle functions as a receptor for different bacteria and fungi, leading to proper immune responses that functions to eradicate pathogens (38, 82). Early response mediated *via* TLRs and MCL expressed on macrophages by a primary infectious stimulus (PAMP) results in the upregulation of Mincle expression. This leads to a sustained signaling process *via* the activating motif of the FcRγ chain and the production of pro-inflammatory cytokines and finally a non-specific activation of Th1/Th17 response. In an appropriate immune response, the end product is the eradication of the infectious agent and resolution of the inflammation. Mincle stimulation can help by inducing anti-inflammatory genes and genes involved in wound healing. In case of a sustained infection when pathogenic ligands are still present or because of tissue damage resulting in the presence of DAMPs or self-associated molecular patterns (SAMPs) continuous Mincle signaling remains. TLR- and Mincle co-dependent genes are enriched among genes required to handle persisting D/M/SAMP signals (38).

There are several pathways that counter-regulate Mincle on macrophages and DCs. Among them is the observed effect of IL-4 on Mincle expression of monocyte-derived DCs (87). This is, however, an artificial system, because these cells co-express surface markers (CD83 and DC-SIGN) that are not found to on the same cells in the *in vivo* situation (88).

In CD, there is evidence that a dysregulated macrophage function and a consecutive defective acute inflammatory response result in the impaired clearance of commensal bacteria. The persistence of the bacteria leads to a chronic granulomatous inflammation. Pathogenic infections may act as triggers or contributing factors for the chronic inflammation (2, 89) that is mediated by other stimuli of various nature, involving microbial-associated molecular patterns, DAMPs, or SAMPs, all described to be ligands of Mincle.

It will be of potential interest to study the direct role of Mincle as a predominant activating C-type lectin receptor *via* a Syk/Card9-dependent signaling mechanism in CD. Genetic susceptibility, barrier defects, or bacterial handling, dysbiosis or infection, sustained innate immunity, and defective regulation are all layers of a multi-hit model of intestinal inflammation (2). They are combined with different homeostatic modules such as autophagy, ER stress, antimicrobial proteins, the microbiota, PRRs, cytokine modules, and regulatory T cells. Defective modules may predispose people to the development of chronic intestinal inflammation. Determination of the role of Mincle in these layers and modules will reveal if Mincle is an important receptor of mediator of the chronic nature of CD, which could be relevant for therapeutic intervention. Targeting Syk has been suggested as a treatment for allergic and autoimmune disorders (90). In rheumatoid arthritis, inhibition of Syk has been studied as a treatment option (91). Although there is no direct evidence on the role of Mincle in CD, the data on the expression and role of Mincle in health and disease reveal numerous potential starting points. The synergy and antagonisms of the various PRRs, whether these are C-type lectins or TLRs, and their differential regulation on cells of the innate immune system, macrophages, and DCs is an important topic to understand the endogenous adjuvant reaction that they might induce. There are probably multiple mechanisms and interactions that result in the observed pathogenic immune reaction that is the fundament of CD. Here, the surprising overlap between features of CD and the roles that Mincle plays in a (chronic) immune reaction might indicate that CD could be an adjuvant reaction induced by Mincle triggering.

## Author Contributions

The author confirms being the sole contributor of this work and approved it for publication.

## Conflict of Interest Statement

The author declares that the research was conducted in the absence of any commercial or financial relationships that could be construed as a potential conflict of interest.
